# Macrophage polarization by MSC-derived CXCL12 determines tumor growth

**DOI:** 10.1186/s11658-021-00273-w

**Published:** 2021-06-26

**Authors:** Shabnam Babazadeh, Seyed Mahdi Nassiri, Vahid Siavashi, Mohadeseh Sahlabadi, Mostafa Hajinasrollah, Mohamad Zamani-Ahmadmahmudi

**Affiliations:** 1 Department of Clinical Pathology, Faculty of Veterinary Medicine, University of Tehran, Tehran, Iran; 2 Department of Stem Cells and Developmental Biology at Cell Science Research Center, Royan Institute for Stem Cell Biology and Technology, ACECR, Tehran, Iran; 3 Department of Clinical Science, Faculty of Veterinary Medicine, Shahid Bahonar University of Kerman, Kerman, Iran

**Keywords:** Mesenchymal stem cells (MSCs), Macrophage, Cytokine, C-X-C motif chemokine ligand 12 (CXCL12), Phagocytosis, Tumor microenvironment

## Abstract

**Background:**

Phenotypic and functional heterogeneity of macrophages is known to be the main reason for their ability to regulate inflammation and promote tumorigenesis. Mesenchymal stem cells (MSCs) are one of the principal cells commonly found in the tumor stromal niche, with capability of macrophage phenotypic switching. The objective of this study was to evaluate the role of C-X-C motif chemokine ligand 12 (CXCL12) produced by marrow-derived MSCs in the phenotypic and functional pattern of bone marrow-derived macrophages (BMDMs).

**Methods:**

First, the CRISPR/Cas9 system was used for the CXCL12 gene knock-out in MSCs. Then, coculture systems were used to investigate the role of MSCs^*CXCL12−/−*^ and MSCs^*CXCL12+/+*^ in determination of macrophage phenotype. To further analyze the role of the MSC-derived CXCL12 niche, cocultures of 4T1 mammary tumor cells and macrophages primed with MSCs^*CXCL12−/−*^ or MSCs^*CXCL12+/+*^ as well as in-vivo limiting dilution assays were performed.

**Results:**

Our results revealed that the expression of IL-4, IL-10, TGF-β and CD206 as M2 markers was significantly increased in macrophages co-cultured with MSCs^*CXCL12+/+*^ , whereas the expression of IL-6, TNF-α and iNOS was conversely decreased. The number and size of multicellular tumor spheroids were remarkably higher when 4T1 cells were cocultured with MSC^*CXCL12+/+*^-induced M2 macrophages. We also found that the occurrence of tumors was significantly higher in coinjection of 4T1 cells with MSC^*CXCL12+/+*^-primed macrophages. Tumor initiating cells were significantly decreased after coinjection of 4T1 cells with macrophages pretreated with MSCs^*CXCL12−/−*^.

**Conclusions:**

In conclusion, our findings shed new light on the role of MSC-derived CXCL12 in macrophage phenotypic switching to M2, affecting their function in tumorigenesis.

**Supplementary Information:**

The online version contains supplementary material available at 10.1186/s11658-021-00273-w.

## Background

Tissue macrophages are members of the mononuclear phagocyte system, signifying their ontogeny and phagocytic activity. Great functional and phenotypic diversity was reported within these cells. Stage-specific functional dynamism of macrophages ranging from injury initiation to inflammation and finally instructing repair is crucial for tissue homeostasis [1–3]. It is now clear that this developmental dynamism results from a complex interplay between intrinsic differentiation pathways and environmental signals received from neighboring cells [4]. It has been demonstrated that the functional dynamism of macrophages depends on their phenotypic polarization [5]. Macrophages are able to oscillate between pro-inflammatory M1 and debris-scavenging/tissue-remodeling alternatively activated M2 states [1]. This broad range of functional dynamism results in macrophages being involved in several chronic pathologic conditions within the tumor environment [6]

It has been reported that macrophages represent a prominent component (up to 50% of cells) of infiltrated leukocytes in malignancies [7]. The presence of M2 macrophages has been correlated with worse clinical consequences in a series of neoplastic conditions [8, 9].

Therefore, recognition of macrophage polarization and biological factors involved in this process has now become the area of many investigations. Hypoxia and the various signals derived from stromal niche cells profoundly regulate macrophage polarization [4]. Generally interferon gamma, lipopolysaccharide, tumor necrosis factor (TNF) and granulocyte–macrophage colony-stimulating factor are known as M1 inducers while colony stimulating factor-1, IL-4, IL-13 and IL-10 and hypoxia are involved in M2 polarization [10].

Mesenchymal stromal cells (MSCs) are immature, adherent stromal cells residing in various tissues and commonly found at injury sites and in tumors [11]. Emerging data suggest that MSCs can promote tumorigenic processes, including neoplastic tissue formation, maintenance, and chemoresistance, as well as tumor growth [11, 12]. Some recent evidence demonstrated that significant functional interactions can occur between MSCs and macrophages, such that MSCs regulate the function of macrophages and induce macrophage differentiation/polarization via secreting various types of chemokines [13–15].

Among MSC-released chemoattractants, C–X–C motif chemokine ligand 12 (CXCL12), also known as stromal cell derived factor 1 (SDF-1), is known to be highly inducible in some pathologic states involving ischemia or hypoxia and in proangiogenic environments, such as tumors and wounds [16–18]. CXCL12 is a small molecule with immunomodulatory properties as it is a highly effective lympho-monocyte chemoattractant [6, 17, 19–21]. A series of G protein-coupled chemokine receptors, which are characterized by seven transmembrane domains, can be activated by the N-terminus part of CXCL12. CXCR4, a 352-amino acid molecule, is the main receptor for CXCL12, which transduces multiple signals after induction, resulting in a number of context-dependent biological functions such as proliferation, cell chemotaxis, migration, apoptosis, survival and differentiation [17, 22–24].

Sánchez-Martín et al. reported that monocyte-derived CXCL12 led monocytes to differentiate into a distinct subtype of macrophages, characterized by enhanced expression of CD14 and CD163 and the secretion of proangiogenic factors, such as vascular endothelial growth factor and CCL1 [16]. Moreover, CXCL12 in the tumor microenvironment was reported to induce macrophage mobilization and vasculogenesis [21].

Considering the importance of macrophage polarization with subsequent functional changes in different physiopathological conditions, and with respect to the role of MSC as an effector cell in macrophage polarization dynamism, this study was designed to evaluate the role of MSC-derived CXCL12 in macrophage polarization and phagocytosis. In this regard, we applied the CRISPR/Cas9 system to knock out the CXCL12 gene in bone marrow (BM)-derived MSCs in order to discover the biological effect of the MSC-derived CXCL12 niche on BM macrophages.

## Methods

### Animals

Eight-week-old female BALB/c mice were used in this study. The mice were treated according to the *Guide* *for* *the* *Care* *and* *Use* *of* *Laboratory* *Animals* (NIH Publication, 8th edition, revised 2011). All steps of this study received consent from the Animal Care Committee of the University of Tehran.

### Cells and culture conditions

Isolation and culture of BM macrophages and MSCs were performed using standardized protocols with some modifications [25–28]. Briefly, we flushed the femurs of mice with PBS and harvested the bone marrow mononuclear cells (BM-MNCs) by centrifugation on a Ficoll (Cat No: 10771; Sigma Aldrich, USA) gradient. Afterwards, the total cell count was carried out using a cell counter machine (Model: MEK-6450k, Nihon Kohden; Japan). For expansion of bone marrow derived macrophages (BMDMs), MNCs were cultured in RPMI-1640 containing 10% FBS, 3% penicillin–streptomycin and 7.5 mL of L929 cell-conditioned medium at 37 °C, 5% CO2, and 95% relative humidity for one week. Cells were washed and passaged after 70% confluency [13].

For the purpose of MSC expansion, BM-MNCs were plated into 24-well dishes and cultured in Dulbecco's modified Eagle's medium (DMEM) (Cat. No: 11-995-040; Gibco) high glucose, supplemented with 10% FBS and 3% penicillin–streptomycin at 37 °C, 5% CO_2_, and 95% relative humidity. Cells of the third passage were used for our experiments. 

For experimental tumor induction, the 4T1 breast cancer cell line (which is a triple-negative breast cancer tumor-initiating cell (TNBC-TIC) with similar aggressive phenotype to the human disease) were purchased from ATCC. Cells were cultured in DMEM supplemented with 10% fetal bovine serum (FBS) at 37 °C humidified atmosphere with 5% CO_2_. Cells were then washed and passaged at almost 80% confluency [29].

### Flow cytometry and characterization

Flow cytometric analysis was performed to characterize cultured MSCs and macrophages isolated from bone marrow. For this work, cells were incubated with 5% BSA solution at 4 °C for 20 min. MSCs were then incubated with a panel of antibodies, including PE-conjugated anti-mouse CD34 (Cat. No: ab187284; Abcam, Inc.), PE-conjugated anti-mouse CD45 (Cat. No.: 12-0451-81; eBioscience, Inc.), PE-conjugated anti-mouse CD105 (Cat. No.: 120407; BioLegend, Inc.), PE-conjugated anti-mouse CD90 (Cat. No.: 12-0900-81; eBioscience, Inc.), and macrophages were incubated with PE-conjugated anti-mouse F4/80 (Cat. No.: ab105156; Abcam) at 4 °C for 30 min [30]. Ultimately, the cells were washed twice with PBS and analyzed by BD FACSAria II (BD Biosciences). For CD206 staining, Alexa Fluor 488-conjugated anti-mouse CD206 (Cat. No.: 141709; BioLegend Inc.) was used. In flow cytometry analyses, cell viability was first performed by staining cells with 7-AAD or DAPI. Data were analyzed using FlowJo v7.6.5 software.

### CXCL12 gene knock-out in MSCs

The CRISPR/Cas9 system was utilized for knocking out the CXCL12 gene in BM-derived MSCs. Accordingly, we seeded 2 × 10^5^ cells in a 6-well culture plate and incubated in DMEM containing 10% FBS. After 60–80% confluency, we transfected the cells with CXCL12 CRISPR/Cas9 KO Plasmid (Cat No: sc-422854, Santa Cruz, Inc. USA) using a commercial transfection reagent (Cat No: sc-395739, Santa Cruz, Inc. USA) in accordance with the manufacturer's instructions. Transfected cells expressing plasmids were recognized by emergence of green fluorescent protein (GFP) signals resulting from a GFP reporter gene in the plasmid. Western blot analyses were performed to confirm the CXCL12 gene knock-out in MSCs. A mock plasmid was used in our transfection experiments as a negative control.

### Co-cultures of BMDMs and MSCs

For transwell co-cultures, BMDMs were seeded into a 24-well plate. The next day, 0.4-mm-pore size Corning Transwell inserts (Cat. No: 35324; SPLInsert) containing 2 × 10^5^ MSCs^*CXCL12−/−*^ or 2 × 10^5^ MSCs^*CXCL12+/+*^ were placed into the macrophage containing plates. After 48 h incubation, macrophages were harvested for a series of experiments to investigate the phenotype switching by ELISA tests, western blotting and flow cytometry.

### Enzyme-linked immunosorbent assay (ELISA)

The conditioned medium from each culture was centrifuged at 6000*g* for 5 min at 4 °C to exclude cell and cell debris contamination. The level of IL-10/IL4/IL6/ transforming growth factor beta (TGF-β) / tumor necrosis factor alpha (TNF-α) in the conditioned medium was quantified using Quantikine ELISA kits for mouse IL-10 (Cat No: DY417; R&D), IL-4 (Cat No: M4000B; R&D), IL-6 (Cat No: M6000B; R&D), TGF-β (Cat No: DY1679; R&D), and TNF-α (Cat No: MTA00B; R&D) according to the manufacturer’s instructions.

### Western blotting

Cells to be subjected to western blot analysis were lysed in a lysis buffer (Cat No: R0278, Sigma Aldrich, USA). Proteins were quantified using the Bradford assay. We relocated lysates (20 μg) to SDS-PAGE after 5 min boiling and after that transferred them to 0.2 μm Immun-Blot polyvinylidene difluoride membranes (Cat No: 162-017777; Bio-Rad Laboratories, CA, USA). Next, the membranes were blocked with 3% non-fat dry milk (Cat No: 1.15363.0500; Merck KGaA, Darmstadt, Germany) in Tris-buffered saline containing 0.1% Tween 20 (TBST) for 1 h. Then, we incubated the membranes with primary antibodies, including anti-CXCL12 (Cat No: sc-28876, Santa Cruz, Inc. USA), anti-inducible nitric oxide synthase (iNOS) (Cat No: sc-28876, Santa Cruz, Inc. USA) and anti-β actin-loading control (Cat No: ab8224; Abcam) at room temperature for 1 h, washed them three times with TBST, and afterward incubated them with HRP-conjugated secondary antibody, which is a rabbit anti-mouse IgG-HRP (1:4000; Cat No: ab6728; Abcam), for 1 h at room temperature. The membranes were incubated with enhanced chemiluminescence (ECL) for 1–2 min. Protein expression was normalized to β-actin. Densitometry of protein bands was performed using the gel analyzer Version 2010a software (NIH, USA), so that the percentage area under the curve of each band was divided by the percentage area under the curve of its corresponding actin band, and then calculated values were compared between groups.

### Phagocytosis assay

Fluorescein isothiocyanate- (FITC-) conjugated dextran (Cat. No: 68059; Sigma Aldrich, USA) was used to determine the phagocytic function of macrophages. Bone marrow macrophages co-cultured with MSCs^*CXCL12+/+*^ or MSCs^*CXCL12−/−*^ were adjusted to a concentration of 3 × 10^5^ cells in 100 μL of complete RPMI 1640 medium and pre-incubated on ice for 30 min. Then, the above cells were incubated with 20 mg/L dextran-FITC for 30 min at 37 °C or at 4 °C to detect nonspecific binding. Cells were washed three times with 500 μL of complete RPMI 1640 medium and fixed in 10% (vol/vol) formaldehyde-PBS. Median fluorescence intensities (MFIs) and the percentage of dextran-positive cells were determined by flow cytometry using the BD FACSAria II system (BD Biosciences).

### Three-dimensional co-cultures

For 3D co-cultures, a commercially available polysaccharide hydrogel system (Cat. No: TWG001; Vitrogel—TheWell Bioscience) which mimics the natural ECM (extracellular matrix) was used. MSC^*CXCL12+/+*^ and MSC^*CXCL12−/−*^-primed macrophages harvested from the bone marrow were stained with CFSE (Cat. No: 65-0850; Thermo Fisher, Scientific). For this purpose, 1 × 10^5^ cells were incubated in 1 µM dye solution for 10 min at room temperature in the dark. 4T1 cells were also labeled with CM-DiI (Cat. No: V-22888; Molecular probes) at 5 µM concentration according to the manufacturer’s instructions. After labeling, 4 × 10^4^ macrophages (MSC^*CXCL12+/+*^ or MSC^*CXCL12−/−*^*-*primed macrophages) were mixed with 4 × 10^4^ 4T1 cells in a final volume of 40 µl of culture medium (DMEM/F12). The Vitrogel solution was first diluted with distilled water (DW) at a 1:4 (V/V) ratio. Then, the cell mixture (40 µl) was mixed with 160 µl of diluted Vitrogel and the final mixture was transferred to a 96-well culture plate with 50 µl in each well. The plate was then incubated for 15 min at room temperature for soft gel formation. Finally, the culture medium was added to cover the cell-containing hydrogel and incubated at 37 °C/95% O_2_/5% CO_2_ with more than 90% humidity. Spheroid formation was assessed under an inverted microscope after 72 h.

In order to perform the quantitative evaluation of multicellular tumor spheroids (MCTS), we used the Image Processing toolbox in the MATLAB software. For this purpose, image segmentation was implemented, where MCT images were divided into two segments, including red pixels and background pixels. As the images were in true color RGB with three color bands, namely red, green and blue, the first step was to convert the images to grayscale. Each pixel then had three grayscales corresponding to its color band. By considering three thresholds for each color band, a binary mask image was created, as shown in Fig. 5C. The pixels with a value of 1 in the mask images represent red pixels and those with a value of 0 correspond to background pixels. Considering the corresponding array of the mask images, the sum total of the array values equals the number of all red pixels in each image. To implement the process, a script was written and run in the software.

### Tumor challenge

Twelve 8-week-old female BALB/c mice were divided into three groups in which a mixture of 4T1 cells (1 × 10^5^, 2 × 10^5^ or 4 × 10^5^ cells) and MSC^*CXCL12-/–*^primed macrophages (1 × 10^5^ cells) was injected into the right inguinal mammary fat pads in 10 µL of Matrigel (Cat No: 354248; Corning). For each mouse a corresponding mixture of 4T1 cells (1 × 10^5^, 2 × 10^5^ or 4 × 10^5^ cells) and MSC^*CXCL12+/+*^-primed macrophages (1 × 10^5^ cells) was also injected into the left inguinal mammary fat pads in Matrigel. Tumor development was checked every day. After two weeks, the mice were sacrificed by carbon dioxide asphyxiation and the mammary tumors excised from the mice. We then measured tumor dimensions with a caliper to calculate the tumor volume using the following formula: 0.52 × long diameter × short diameter2 [31].

### Statistical analysis

Data are represented as mean ± SD. Student's t-test was used for statistical analyses after confirming the homogeneity of variances and normal distribution of data. For tumor initiation assay Fisher's exact test and the Cochran–Mantel–Haenszel (CMH) test were used. Statistical analyses were conducted using GraphPad InStat software version 2.02. Values of *P* < 0.05 were considered statistically significant.

## Results

### Phenotypic polarization of BMDMs by MSC-derived CXCL12

**Fig. 1:**
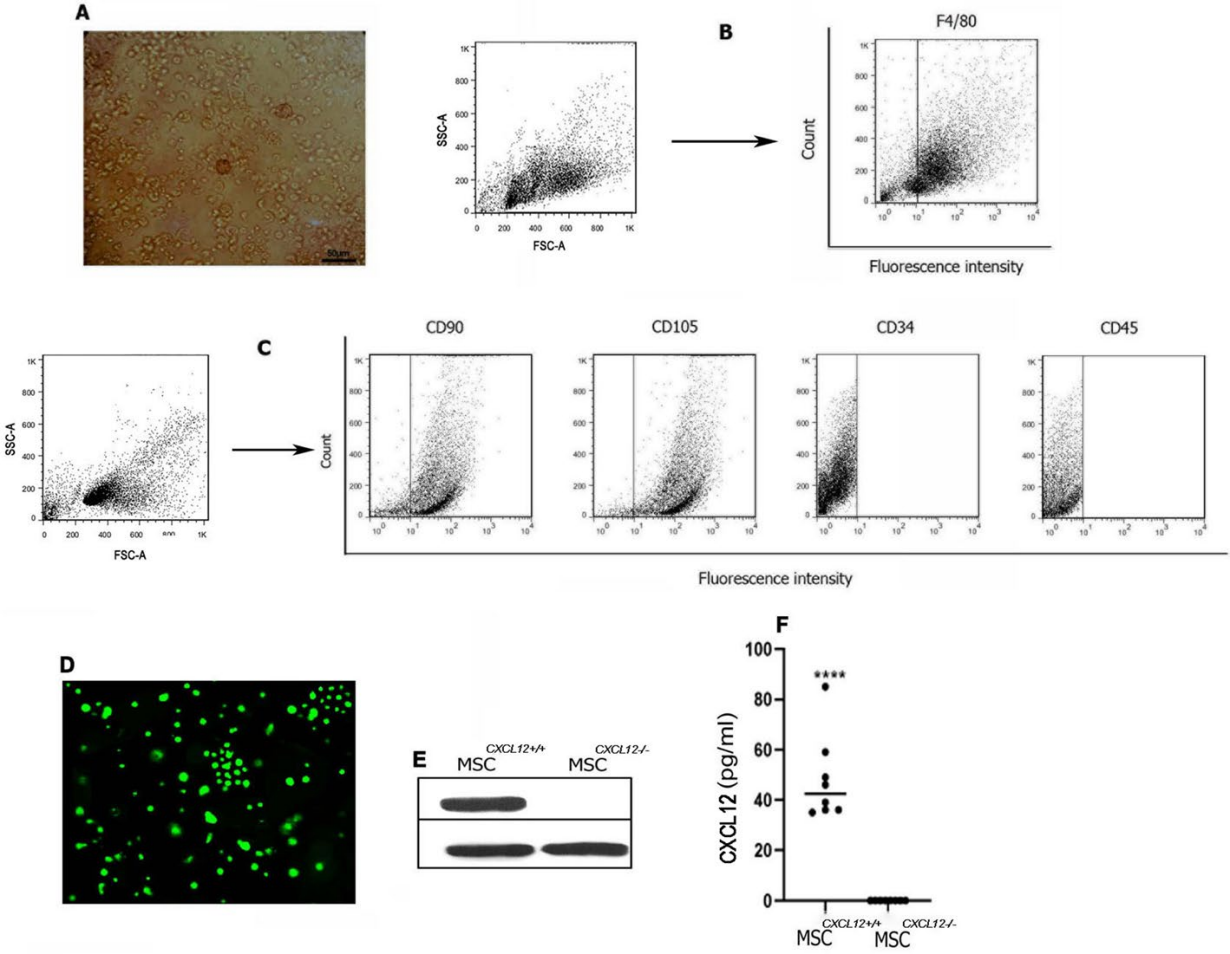
Cell characteristics of bone marrow derived macrophages (BMDMs) and mesenchymal stem cells (MSCs). Phase contrast microscopic image of BMDMs 5 days after in vitro culture (**A**). Flow cytometric analysis of BMDMs for F4/80 (**B**). Flow cytometric analysis of MSCs for CD90, CD105, CD34, and CD45 (**C**). CXCL12 gene knockout in MSCs. Transfection of MSCs with a GFP-positive CRISPR/Cas9 plasmid to knock out CXCL12 gene (**D**). Western blotting for knockout efficiency (**E**). β-actin was used as a control. CXCL12 measurement in the serum-free conditioned media of MSC^*CXCL12+/+*^ and MSC^*CXCL12-/-*^ by ELISA (**F**). Each point indicates an experiment (horizontal lines: mean)

Macrophages and MSCs were expanded from the BM mononuclears. BMDMs were morphologically round with eccentric nuclei and foamy cytoplasm (Fig. 1A). Macrophages were characterized as cells with F4/80 expression [32, 33] (Fig. 1B). MSCs were found to be positive for the expression of CD90 and CD105 and negative for CD34 and CD45 (Fig. 1C). CXCL12 chemokine was found to be expressed by MSCs. We first knocked out the CXCL12 gene in MSCs using a mouse CRISPR-Cas9 CXCL12 knockout system. As shown in Fig. 1D, the cells were efficiently transfected with the GFP reporter containing the CRISPR/Cas9 vector, with transfection efficiency of more than 90%. Furthermore, CXCL12 knockout was confirmed in MSCs by western blotting, as there was no CXCL12 protein expression in these cells (Fig. 1E; Additional file 1: Fig. S1). CXCL12 secretion was measured in the serum-free conditioned media of MSCs^*CXCL12+/+*^ whereas CXCL12 was undetectable in the conditioned media of MSCs^*CXCL12−/−*^ (Fig. 1F). There was no morphological difference in MSCs^*CXCL12+/+*^ and MSCs^*CXCL12−/−*^ as microscopic analyses consistently revealed the outgrowth of mesenchymal cells with a spindle-like morphology in both MSC^*CXCL12+/+*^ and MSC^*CXCL12−/−*^ populations (data is not shown).

**Fig. 2:**
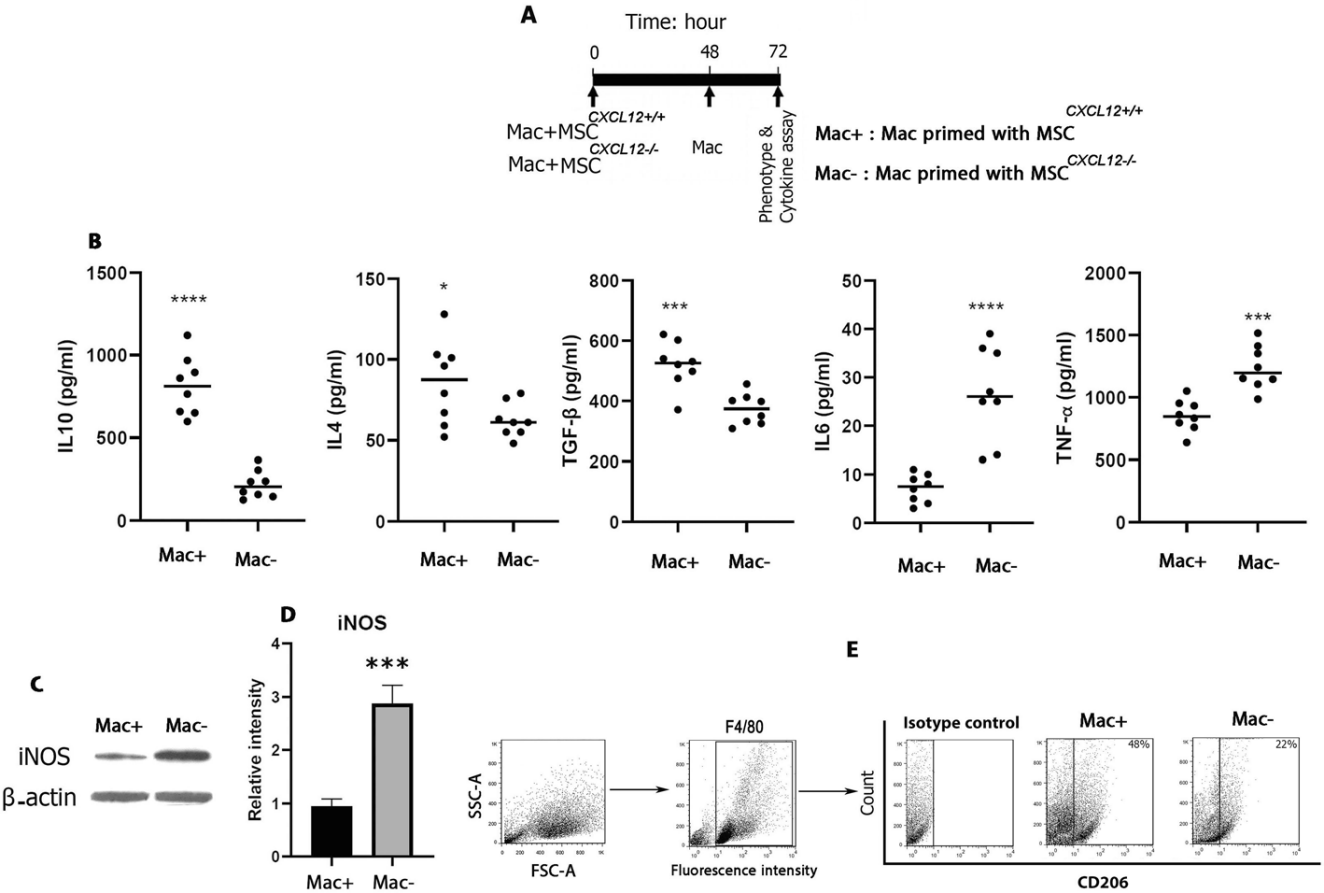
Macrophage phenotypic switching by MSC-derived CXCL12 niche. Schematic diagram of macrophage and MSC cocultures. Bone marrow macrophages were cocultured with MSCs in non-contact Transwell inserts for 48 h. Then, macrophages were incubated for a further 24 h before submission for phenotype and cytokine assays (**A**). Cytokine levels of IL10, IL4, TGF-β, IL6 and TNF-α in supernatants of macrophages after non-contact coculture with MSCs^*CXCL12+/+*^ (M2) or MSCs^*CXCL12−/−*^ (M1). Each point indicates an experiment (horizontal lines: mean) (**B**). Western blot depicting protein levels of iNOS (**C**). Left and right lanes indicate iNOS in Mac in the presence of MSCs^*CXCL12+/+*^ and MSCs^*CXCL12−/−*^, respectively. Representative data from three independent experiments (**C**). Quantitative analysis of iNOS levels (**D**). Representative flow cytometry dot plots of CD206 expression by macrophages after culturing with MSCs^*CXCL12+/+*^ (M2) or MSCs^*CXCL12−/−*^ (M1). MSC-derived CXCL12 niche strongly induced CD206 expression by macrophages (**E**). Representative FSC/SSC and F4/80 gating is shown. Data from three independent experiments with the mean percentage of positive cells in each group are shown within histograms (**E**). *P < 0.5, ***P < 0.001, ****P < 0.0001, Student's t-test. Mac, macrophage

We then assessed the role of the MSC-derived CXCL12 niche in BMDM phenotypic switching in non-contact co-culture systems (Fig. 2A). We measured the protein levels of IL-4, IL-10 and TGF-β as M2 macrophage markers as well as IL-6 and TNF-α as M1 macrophage markers in the macrophage cell culture media by ELISA. Intriguingly, our findings showed that the deletion of CXCL12 from the MSC niche significantly reduced the expression of M2 macrophage markers in the BMDM cells, whereas the M1 macrophage markers were markedly increased (Fig. 2B).

We also evaluated the expression of the surface receptor CD206, a specific M2 macrophage marker, in BMDMs co-cultured with MSCs^*CXCL12+/+*^ or MSCs^*CXCL12−/−*^ using flow cytometry. Our results showed that the percentage of CD206 positive cells was decreased in BMDMs co-cultured with MSCs^*CXCL12−/−*^ (Fig. 2E). Moreover, the expression level of iNOS was remarkably increased in BMDMs co-cultured with MSCs^*CXCL12−/−*^ (Fig. 2C, D; Additional file 2: Fig. S2), further confirming that M1 macrophage phenotypic switching is induced by coculturing with MSCs^*CXCL12−/−*^.

Thus, these results demonstrated that the BM MSC-derived CXCL12 niche was able to induce an M2 phenotype in BMDMs.

### MSC-derived CXCL12 effect on BMDM function

**Fig. 3:**
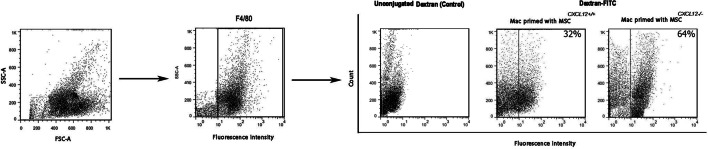
Phagocytic function of MSC^*CXCL12+/+*^-primed macrophages and MSC^*CXCL12−/−*^-primed macrophages. Representative histogram of flow cytometry data showing higher dextran-FITC uptake by MSC^*CXCL12−/−*^-primed macrophages compared with MSC^*CXCL12+/+*^-primed macrophages. Representative FSC/SSC and F4/80 gating is shown. Data from three independent experiments with the mean percentage of positive cells in each group are shown within histograms. Unconjugated dextran was used as a control. Mac, macrophage

We investigated the phagocytic properties of macrophages primed with MSCs^*CXCL12+/+*^ compared with macrophages primed with MSCs^*CXCL12−/−*^. As shown in Fig. 3, macrophages co-cultured with MSCs^*CXCL12−/−*^ exhibited enhanced phagocytic potency compared with MSC^*CXCL12+/+*^-primed macrophages. Our results showed that CXCL12 is a significant inhibitor of macrophage phagocytic activity.

### Augmentary effect on tumor induction by BMDMs primed with MSC-derived CXCL12

**Fig. 4:**
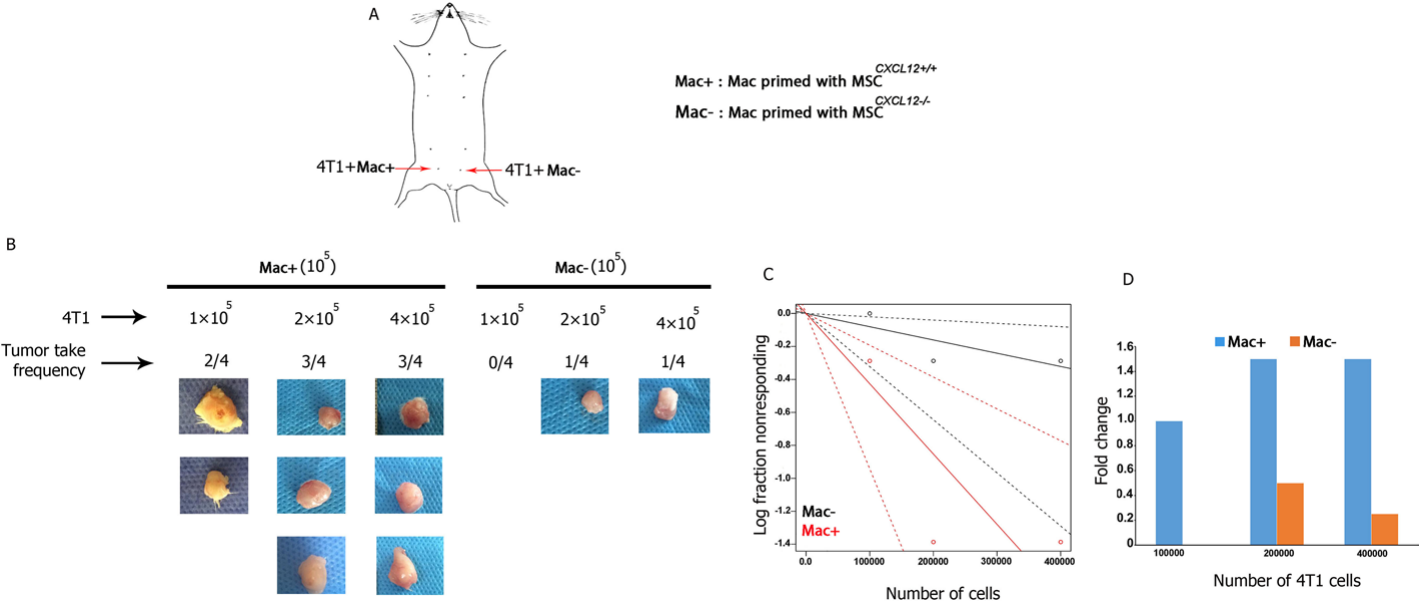
Occurrence of mammary tumor in three independent cellular dilutions of MSC^*CXCL12+/+*^ and MSC^*CXCL12-/–*^primed macrophages. Schematic representation of cell injection experiments (**A**). In vivo limiting dilution assay was performed using 4T1 cells and MSC^*CXCL12+/+*^ and MSC^*CXCL12−/−*^-primed macrophages. Tumor frequency rate and individual tumor masses are shown in each dilution (**B**). Limiting dilution analysis using ELDA software to estimate the frequency of the tumor-initiating cells by fitting the single-hit Poisson model (SHPM). In this plot, regression lines and 95% confidence intervals for both groups were graphed (**C**). Average tumor occurrence of 4T1 cells coinjected with three dilutions of MSC^CXCL12+/+^ and MSC^*CXCL12-/–*^primed macrophages (**D**). The Y axis was generated based on the rationale that the occurrence of mammary tumor in one mouse/100 000 injected tumor cells was arbitrarily assumed as 1. Mac, macrophage

To assess whether BMDM phenotypic switching by MSC-derived CXCL12 might have an impact on tumor induction by 4T1 cells, a mixture of 4T1 cells plus BMDM co-cultured with MSCs^*CXCL12+/+*^ or MSCs^*CXCL12−/−*^ was respectively injected into the left and right inguinal mammary fat pads (four fat pads in each group) (Fig. 4A). For this purpose, different numbers of 4T1 cells (1 × 10^5^, 2 × 10^5^, 4 × 10^5^) were co-injected with 1 × 10^5^ macrophages into each fat pad.

Our results showed remarkably augmented tumor induction potency by 4T1 cells after co-injection with MSC^*CXCL12+/+*^-primed macrophages. While coinjection of 1 × 10^5^ 4T1 cells with MSC^*CXCL12−/−*^*-*primed macrophages precluded orthotropic tumor induction, coinjection of similar 4T1 cell numbers with MSC^*CXCL12+/+*^-primed macrophages resulted in tumor induction in two out of four mammary fat pads (mean tumor size, 217 mm3) (Fig. 4B). While the tumor induction rate was three out of four fat pads with co-injection of higher concentrations of 4T1 cells (2 × 10^5^ or 4 × 10^5^) and MSC^*CXCL12+/+*^-primed M2 macrophages (mean tumor size in the 2 × 10^5^ group, 103 mm3; mean tumor size in the 4 × 10^5^ group, 347 mm3), these concentrations of 4T1 cells had a tumor induction rate of just one out of four fat pads after co-injection with MSC^*CXCL12−/−*^*-*primed M1 macrophages with tumor sizes of 27 mm3 and 125 mm3 in the 2 × 10^5^ and 4 × 10^5^ groups, respectively (Fig. 4B).

The results of in vivo limiting dilution assay using ELDA software are depicted in Fig. 4C. Furthermore, the tumor-initiating cell frequencies were calculated as 23 450 (CI = 10 613–51 814) and 124 331 (CI = 31,020–498,340) in MSC^*CXCL12+/+*^-primed M2 and MSC^*CXCL12−/−*^*-*primed M1 macrophage groups, respectively.

Our analysis using Fisher's exact test and the CMH test revealed that occurrence of tumors was significantly higher in the MSC^*CXCL12+/+*^-primed group than the MSC^*CXCL12−/−*^*-*primed group (chi-squared = 6.54, *P* = 0.011). Considering the occurrence of tumor in a single mouse/100 000 injected cells as one fold, average occurrences of tumor across three independent experiments in MSC^*CXCL12+/+*^-primed and MSC^*CXCL12−/−*^*-*primed macrophage groups were 1.3 and 0.25 fold, respectively (Fig. 4D).

**Fig. 5:**
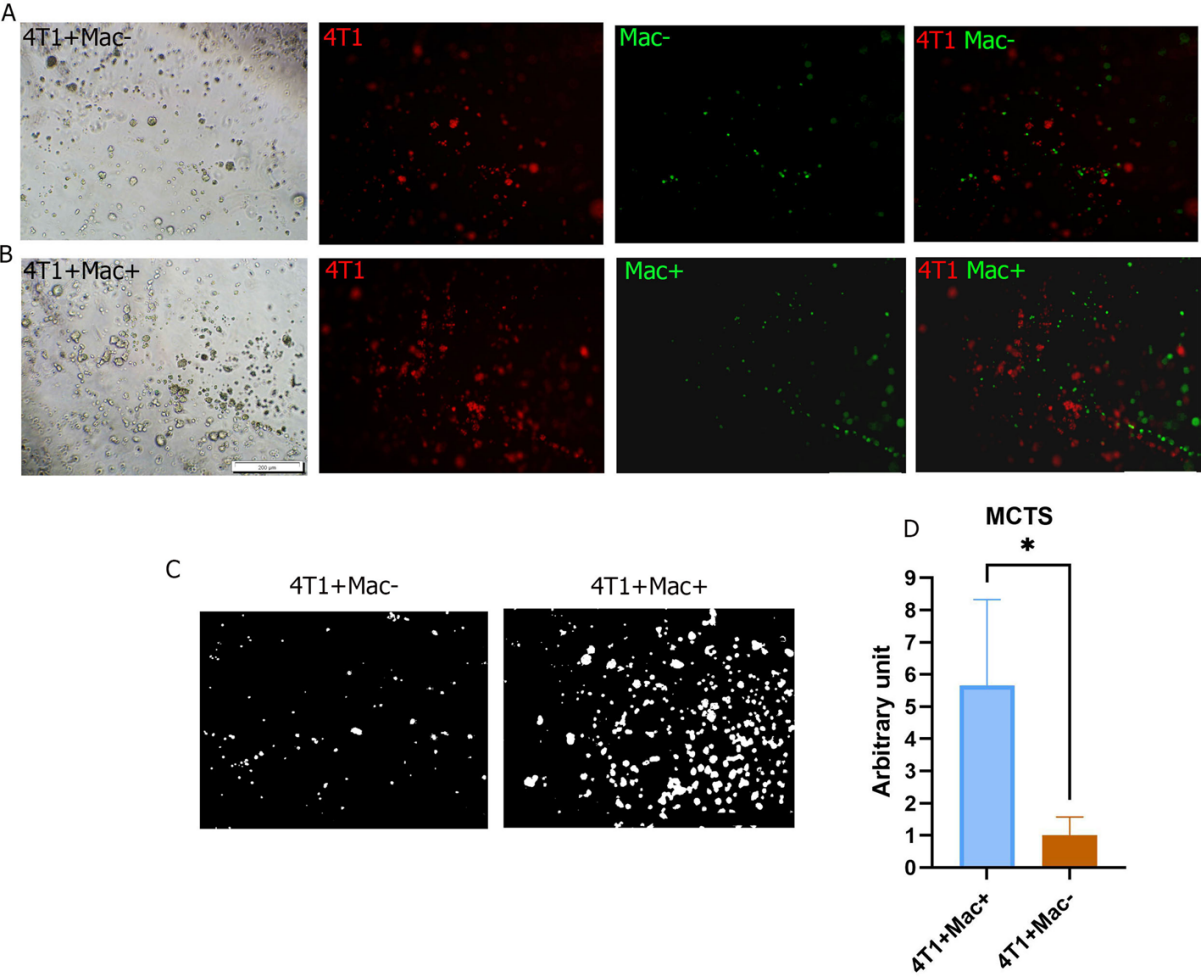
Multicellular tumor spheroids (MCTS) by 4T1 cells after 72 h coculture with MSC^*CXCL12−/−*^-primed (**A**) and MSC^*CXCL12+/+*^-primed macrophages (**B**). 4T1 tumor cells and macrophages were labeled with CM-DiI and CFSE, then cocultured in the Vitrogel matrix. Representative mask images of MCTS by 4T1 cells in each group produced by the MATLAB software (**C**). The number of 4T1 colonies and the size of tumor spheroids were greater after coculturing 4T1 cells with macrophages primed with MSCs^*CXCL12+/+*^ (**D**). *P < 0.5, Student's t-test. Mac, macrophage

Next, we confirmed BMDM phenotypic switching by three-dimensional coculture of 4T1 and MSC^*CXCL12−/−*^ or MSC^*CXCL12+/+*^-primed macrophages. Spheroid formation was assessed under an inverted microscope after 72 h (Fig. 5A, B). Multicellular tumor spheroids (MCTS) were formed one day after culture and rapidly increased in size and number. Three days after co-culturing 4T1 cells with MSC^*CXCL12+/+*^-primed macrophages, large MCTS of 4T1 cells were noted with a scattered population of CFSE-labeled macrophages within the gel (Fig. 5B). A number of macrophages were seen in close association with MCTS. In order to quantitatively evaluate the differences between groups, image processing was performed in the MATLAB software (Fig. 5C). According to the obtained results, the number and size of 4T1 MCTS were remarkably lower in 4T1-MSC^*CXCL12−/−*^-primed macrophage co-cultures than in 4T1-MSCs^*CXCL12+/+*^-primed macrophage co-cultures (Fig. 5D).

## Discussion

In this study, we focused on the effect of CXCL12 produced by BM-MSCs on BMDMs. As polarized macrophages differ in terms of receptor expression, chemokine production and effector functions [4], to observe the effect of MSC-derived CXCL12 on BMDM phenotype, we analyzed secretion of phenotype-specific cytokines, expression of a phenotype-specific CD marker, spheroid formation rate in three dimensional coculture assays and tumor initiation rate after in vivo tumor induction. Our findings revealed that BMDM underwent phenotypic switching into M2 cells following co-culture with MSC^*CXCL12+/+*^, which was documented by augmented expression of IL-4, IL-10, TGF-β, and CD206 as M2 markers, and decreased expression of M1 specific markers, such as IL-6, TNF-α and iNOS. Macrophage M2 phenotype is associated with tumor initiation, development and progression, so that tumor-associated macrophages (TAMs) are recognized as M2 macrophages [9].

Hence, we confirmed our in vitro findings by a three dimensional coculture assay and an in vivo experiment assessing tumor induction rate. According to these tests, the number and size of MCTS were much higher in 4T1 cells co-cultured with MSC^*CXCL12+/+*^-primed (M2) macrophages. Likewise, tumor induction rate was enhanced when 4T1 cells were co-injected into the mice fat pads with these MSC^*CXCL12+/+*^-primed M2 macrophages.

Our findings showed down-regulation of macrophage phagocytic activity after exposure to the MSC-derived CXCL12 niche. Although the potential effect of the MSC CXCL12 niche on macrophage functions has not been investigated yet, M1 macrophages were reported to have greater phagocytic activity [34, 35].

Clinical studies have shown that macrophages can release cytotoxic molecules, such as reactive nitrogen intermediates, reactive oxygen intermediates, and a migration inhibitory factor during chronic inflammation, leading to DNA mutation and defective p53 activity in the surrounding epithelial cells, thereby predisposing these cells to premalignant transformation and tumor initiation. Moreover, TAMs were able to transmit pro-survival signals to these premalignant cells through production of several cytokines, including TNF-α, IL-1, and IL-6 [36].

Tumor-associated macrophages were also found to regulate tumor cell migration/invasion through the extracellular matrix (ECM). Passing through the ECM is a necessary aspect of cell migration. Macrophages can regulate composition of the ECM by depositing ECM components, including various types of collagens. These cells are also a major source of various proteolytic enzymes such as cathepsins, matrix metalloproteinases, serine proteases and urokinase plasminogen activator. Macrophage-derived proteases degrade the surrounding ECM, providing a suitable matrix for cancer cell invasion. Furthermore, TAMs were found to significantly increase the migration and invasion of cancer cells via overexpression of epidermal growth factor [6, 37].

As a matter of fact, it has recently been demonstrated that there is a correlation between M1/M2 ratio and overall survival of ovarian cancer patients, such that the M1/M2 ratio was positively correlated with overall survival and inversely correlated with cancer stage. Iron removal from tumor cells by M1 macrophages and promotion of the antitumor T1 response and cytotoxic T lymphocyte recruitment to tumors by M1 macrophages were suggested to be the main reasons for improvement of the prognosis of patients with ovarian cancer containing an M1-rich microenvironment [38, 39].

With regard to previous studies, MSCs are considered to be responsible for macrophage phenotypic switching [13, 40], but the underlying mechanisms are poorly clarified. Cardiac adipose tissue-derived MSCs were reported to release IL-6, which stimulates macrophages to release M2 inducer cytokines such as IL-10 and IL-13 [41]. Moreover, Vasandan et al. proposed a mechanistic role for bioactive factors such as MSC-derived prostaglandin E2 in instructing M1-M2 polarization shifts [1]. In another study, umbilical cord-derived MSC was found to alter the phenotype and function of monocyte-derived dendritic cells through lactate-mediated metabolic reprogramming. UC-MSC derived lactate can induce M2-macrophage phenotypic switching in terms of morphology, surface markers, migratory properties and antigen presentation capacity [42].

In spite of the fact that CXCL12 is considered as a critical component of the MSC niche [43–46], as yet, it has not been investigated whether MSC-derived CXCL12 might induce phenotypic alteration of macrophages. Stromal cell-derived factor-1 is a small chemotactic, angiogenic factor [47] that was first identified as a molecule involved in B-cell lymphopoiesis using an expression cloning program [20]. Human and mouse CXCR4 [48] and CXCR7 [49] are known as CXCL12 receptors. CXCR4 expression is found in blood monocytes, neutrophils, lymphocytes, hematopoietic progenitors [20] and tissue macrophages [50], and CXCR7 is induced during monocyte-to-macrophage differentiation [51]. Expression of CXCL12 receptors on immune-system cells may be indicative of CXCL12’s role as an important soluble factor operating on effector cells in inflammatory situations and the tumor microenvironment. Interestingly, previous evidence indicated CXCL12 as a critical mediator for monocyte extravasation [16, 20], regulation of monocyte function, survival and differentiation into macrophages [52] and macrophage phagocytosis enhancement [51].

In conclusion, our results suggest that the MSC-derived CXCL12 niche is involved in macrophage polarization dynamics as it could prompt BMDM phenotypic switching into M2 macrophages, which could have a decisive role in the tumor microenvironment. It is noteworthy that all the immunosuppressive circumstances occurring in the cancer stromal niche can result in suppression of cancer immunity, thereby enabling malignant cells to escape immune surveillance and promote tumor growth and metastasis [11]. Thus, investigation of mechanisms interfering with immune suppression and tumor growth may be of great interest in finding new remedies to control and treat cancers.

## Supplementary Information

**Additional file 1.** Uncropped western blot images corresponding to Fig. 1E.

**Additional file 2.** Uncropped western blot images corresponding to Fig. 2C.

## Data Availability

All data are contained in the article.

## Abbreviations

MSC: mesenchymal stem cell
BMDM: Bone marrow-derived macrophage
MNC: Mononuclear cell
TAM: Tumor-associated macrophage
CXCL12: C–X–C motif chemokine ligand 12
SDF-1: Stromal cell-derived factor-1
iNOS: Inducible nitric oxide synthase
TNF-α: Tumor necrosis factor alpha
TGF-β: Transforming growth factor beta
IL: Interleukin
CD: Cluster of differentiation
CFSE: Carboxyfluorescein succinimidyl ester
MCTS: Multicellular tumor spheroids
ECM: Extracellular matrix
3D coculture: Three dimensional coculture
