# Supplementary figures and images for: Macrophage polarization by MSC-derived CXCL12 determines tumor growth
Source: Cell Mol Biol Lett. 2021 Jun 26;26:30. doi: 10.1186/s11658-021-00273-w (PMC8236206; doi:10.1186/s11658-021-00273-w)

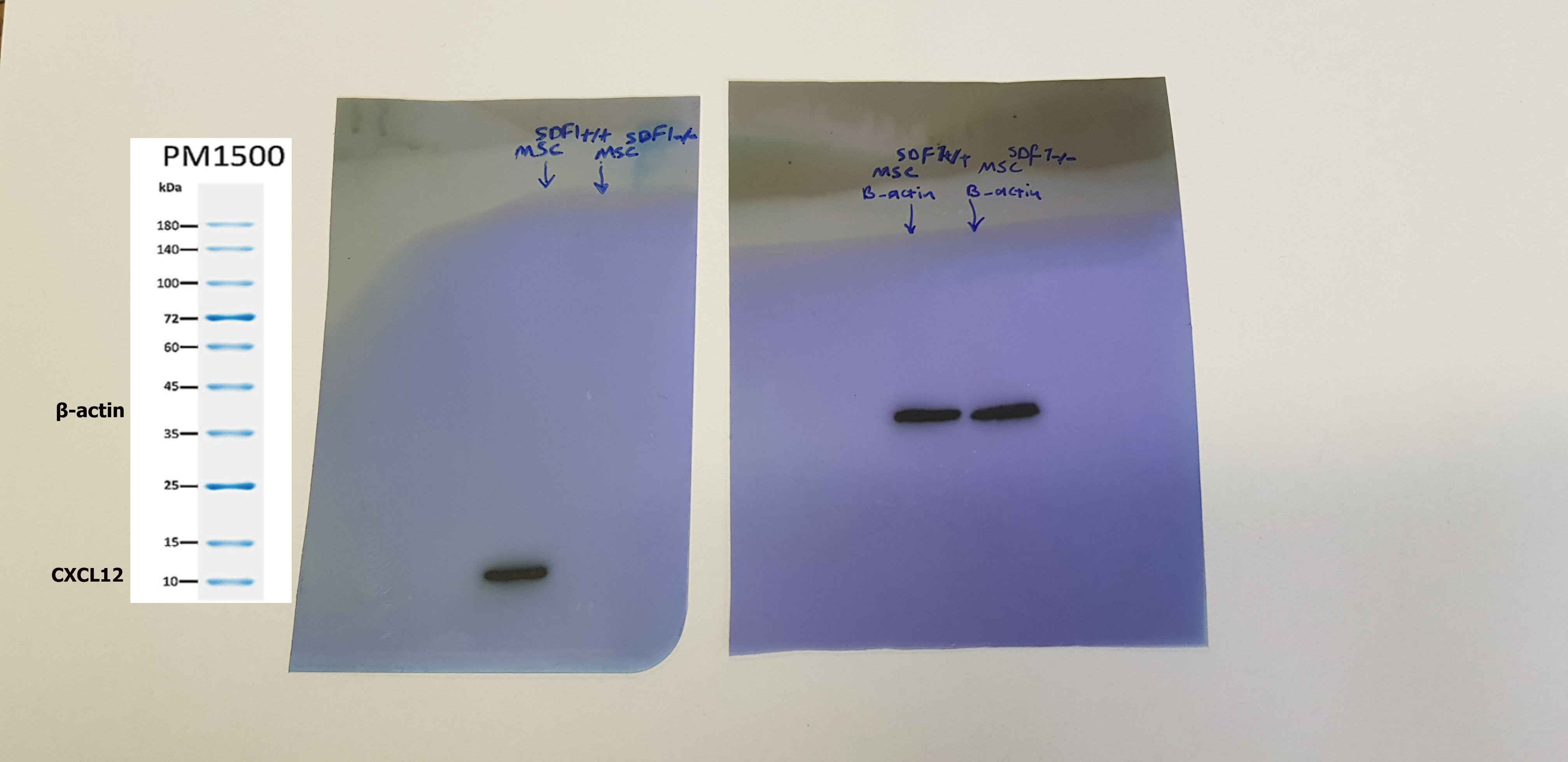

Supplement: Supplementary file 1 — Additional file 1. Uncropped western blot images corresponding to Fig. 1E. [file 11658_2021_273_MOESM1_ESM.tif]

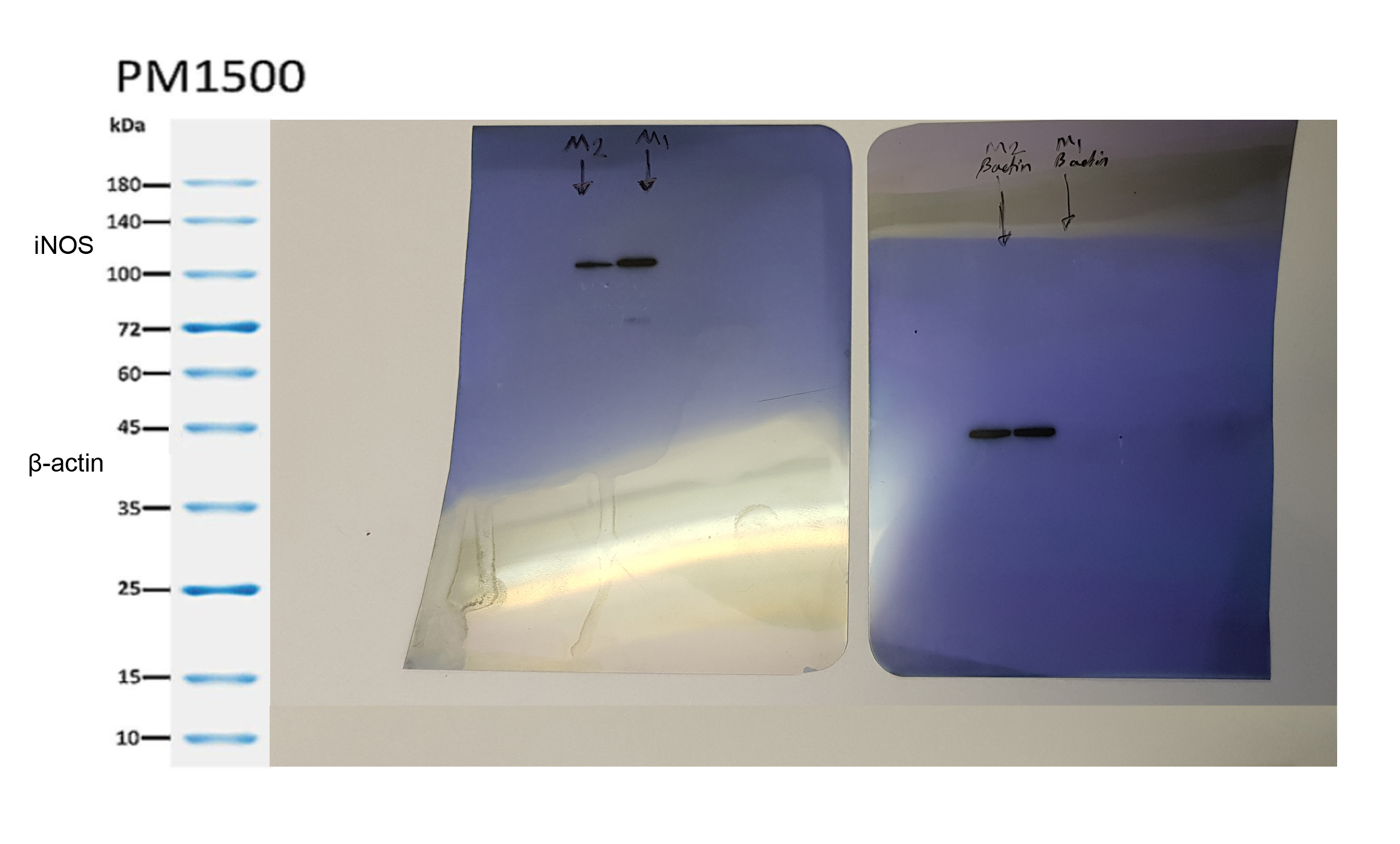

Supplement: Supplementary file 2 — Additional file 2. Uncropped western blot images corresponding to Fig. 2C. [file 11658_2021_273_MOESM2_ESM.tif]
